# Auricular Acupressure for Insomnia in Patients With Maintenance Hemodialysis: A Systematic Review and Meta-Analysis

**DOI:** 10.3389/fpsyt.2021.576050

**Published:** 2021-07-19

**Authors:** Ming Pei, Junli Chen, Shuo Dong, Bo Yang, Kang Yang, Lijuan Wei, Jingbo Zhai, Hongtao Yang

**Affiliations:** ^1^Division of Nephrology, First Teaching Hospital of Tianjin University of Traditional Chinese Medicine, Tianjin, China; ^2^National Clinical Research Center for Chinese Medicine Acupuncture and Moxibustion, Tianjin, China; ^3^Institute of Traditional Chinese Medicine, Tianjin University of Traditional Chinese Medicine, Tianjin, China

**Keywords:** auricular acupressure, insomnia, maintenance hemodialysis, systematic review, meta-analysis

## Abstract

**Background:** Insomnia is one of the common problems in patients with maintenance hemodialysis (MHD). Previous studies have reported the beneficial effects of auricular acupressure (AA) for insomnia in patients with MHD. This study aimed to critically evaluate the efficacy and safety of AA for insomnia in patients with MHD.

**Methods:** Web of Science, Embase, PubMed, Cochrane Library, Chinese Biomedical Database, Wanfang Data, Chinese Science and Technology Periodicals database, and China National Knowledge Infrastructure were systematically searched from inception to April 30, 2020, to identify any eligible randomized controlled trials. MHD patients with insomnia were included regardless of age, gender, nationality, or race. The experimental interventions included AA alone or AA combined with other therapies. The control interventions included placebo, no treatment, or other therapies. The primary outcome was sleep quality measured by the Pittsburgh Sleep Quality Index (PSQI). RevMan 5.3 software was used for statistical analysis.

**Results:** Eight studies involving 618 participants were included for statistical analysis. A meta-analysis showed no significant difference of PSQI global score after 8 weeks of AA treatment compared with estazolam (*p* = 0.70). Other narrative analyses revealed that PSQI global score was significantly attenuated after AA treatment in comparison with mental health education (*p* = 0.03, duration of 4 weeks; *p* = 0.02, duration of 8 weeks), AA plus routine nursing care compared with routine nursing care alone (*p* < 0.0001), and AA plus footbath compared with footbath alone (*p* = 0.01), respectively. A meta-analysis showed that AA could significantly increase the response rate (reduction of PSQI global score by 25% and more) in comparison with estazolam (*p* = 0.01). Other narrative analyses reported that the response rate was significantly increased after AA treatment compared with sham AA (*p* = 0.02), AA compared with mental health education (*p* = 0.04), and AA plus routine nursing care compared with routine nursing care alone (*p* = 0.0003), respectively.

**Conclusion:** The present findings suggest that AA may be an alternative treatment for insomnia in patients with MHD. However, more large-scale, high-quality trials are still warranted to confirm these outcomes.

## Introduction

Insomnia is one of the common problems in patients with maintenance hemodialysis (MHD) (1–3). Insomnia may be caused by a variety of factors, including restlessness, pain, long duration on dialysis, and unhealthy lifestyle behavior (2, 4, 5), which might potentially exert a negative impact on quality of life in dialysis patients (6).

Therapies for insomnia include both pharmacologic (e.g., benzodiazepines, non-benzodiazepine hypnotics) and non-pharmacologic treatments (e.g., cognitive behavioral therapies) (7). However, there is no specific recommendation for insomnia management in MHD patients according to the present clinical practice guidelines (7–10). Moreover, attention should also be paid to the barriers and risks of above interventions. For instance, benzodiazepine treatment may lead to Alzheimer's disease or stroke (11, 12). The effective administration of cognitive behavioral therapy might be limited in dialysis patients due to low patient adherence, lack of trained therapists, etc. (13). Therefore, it is urgently needed to seek alternative treatments.

Complementary and alternative medicine (CAM) has been applied for insomnia worldwide (e.g., in the United States) (14). Auricular acupressure (AA) is defined as a non-invasive technique in CAM to treat certain diseases or to relieve certain symptoms by taping drugs (e.g., semen vaccariae) or magnetic pellets, followed by application of physical pressure at specific acupoints on the ears (15, 16).

Several previous reviews have summarized the efficacy of AA for insomina. For instance, a systematic review indicated that AA might be effective for comorbid insomnia (17). Another meta-analysis showed that AA exerted a beneficial effect on the management of primary insomnia (18). Moreover, a few clinical studies have investigated the efficacy of AA for insomnia in MHD patients. For example, a pilot study revealed that AA could improve sleep quality in MHD patients with severe insomnia (16). Another trial indicated that AA was more effective than sham AA for treating insomnia in MHD patients (19). However, the evidence on AA for insomnia in MHD patients is still inconclusive due to the lack of high-quality systematic reviews.

Therefore, this study aimed to critically evaluate the efficacy and safety of AA for insomnia in patients with MHD.

## Methods

This study was performed according to the Preferred Reporting Items for Systematic Reviews and Meta-Analyses (PRISMA) (20). This study was registered on PROSPERO (No. CRD42020166054).

### Search Strategy

We systematically searched Web of Science, Embase, PubMed, Cochrane Library, Chinese Biomedical Database (CBM), Wanfang Data, Chinese Science and Technology Periodicals database (VIP), and China National Knowledge Infrastructure (CNKI) from inception to April 30, 2020, without restriction on language or publication period. The search terms included (hemodialysis OR haemodialysis OR hemofiltration OR haemofiltration OR hemodiafiltration OR haemodiafiltration OR dialysis) AND (sleep OR insomnia OR wakeful OR sleepless OR “Early Awakening”) AND (auricular OR acupressure OR acupoint OR auriculotherapy) AND (randomized OR randomly OR controlled OR placebo OR trial). Moreover, references of eligible studies were also manually searched to identify additional eligible studies.

### Inclusion and Exclusion Criteria

#### Types of Studies

Randomized controlled trials (RCTs) were included. Quasi-randomized RCTs were excluded.

#### Types of Patients

MHD patients with insomnia were included, without restriction on age, gender, nationality, or race. The diagnosis of insomnia was in line with the internationally accepted diagnostic criteria, such as the 10th edition of International Classification of Diseases (ICD-10) and the Diagnostic and Statistical Manual of Mental Disorders fourth edition-Text Revision (DSM-IV-TR).

#### Types of Interventions

The experimental interventions included AA alone or AA combined with other therapies. The control interventions included placebo, no treatment, or other therapies. The duration and frequency of AA were unrestricted.

#### Types of Outcomes

The primary outcome was sleep quality measured by the Pittsburgh Sleep Quality Index (PSQI). To be specific, PSQI is a self-assessment questionnaire with a global score of 0 to 21 (21), where higher PSQI scores indicate worse sleep quality.

The secondary outcomes included response rate, use of sleep medications, and adverse events. Response was defined based on the reduction of PSQI global score, such as reduction of PSQI global score by 25% or more. Response rate refers to a ratio of the number of patients labeled as “response” divided by the number of all patients in a certain group.

### Selection of Studies

First, titles and abstracts of potentially eligible studies were independently reviewed by two investigators (JC and MP) to delete duplicates and obviously irrelevant publications. Afterwards, full texts of the remaining studies were checked for final inclusion. Discrepancies were resolved through discussion or consultation with another investigator (JZ).

### Data Extraction

The following information was extracted by two investigators (BY and KY): (1) study features (e.g., authors, year of publication, and sample size); (2) patient characteristics (e.g., diagnostic criteria for insomnia); (3) interventions (e.g., frequency, duration of treatment); and (4) outcome measurements (e.g., PSQI, response rate, use of sleep medications, and adverse events). If necessary, we attempted to contact authors by e-mail to obtain missing information.

### Quality Assessment

Cochrane's risk of bias assessment tool was used to evaluate the methodological quality by two independent investigators (SD and MP). It includes seven items: random sequence generation, allocation concealment, blinding of personnel and participants, blinding of outcome measurement, incomplete outcome data, selective reporting, and other sources of bias (22). Risk of bias in each item is categorized into three levels, namely, low, high, or unclear.

### Statistical Analysis

RevMan 5.3 software was utilized for statistical analysis. Risk ratio (RR) with 95% confidence intervals (CIs) was used for the dichotomous variables. Mean difference (MD) with 95% CIs was calculated for the continuous variables. Chi-square test and *I*^2^ statistics were employed to investigate the heterogeneity across studies. In the case of *p* > 0.10 or *I*^2^ <50%, a fixed-effect model was used to estimate pooled effect; otherwise, a random-effect model was used. Moreover, funnel plot was used to examine publication bias of the meta-analysis including 10 or more studies. If necessary, subgroup or narrative analysis was conducted based on the therapeutic duration, comparisons, etc.

## Results

### Literature Search

**Figure 1:**
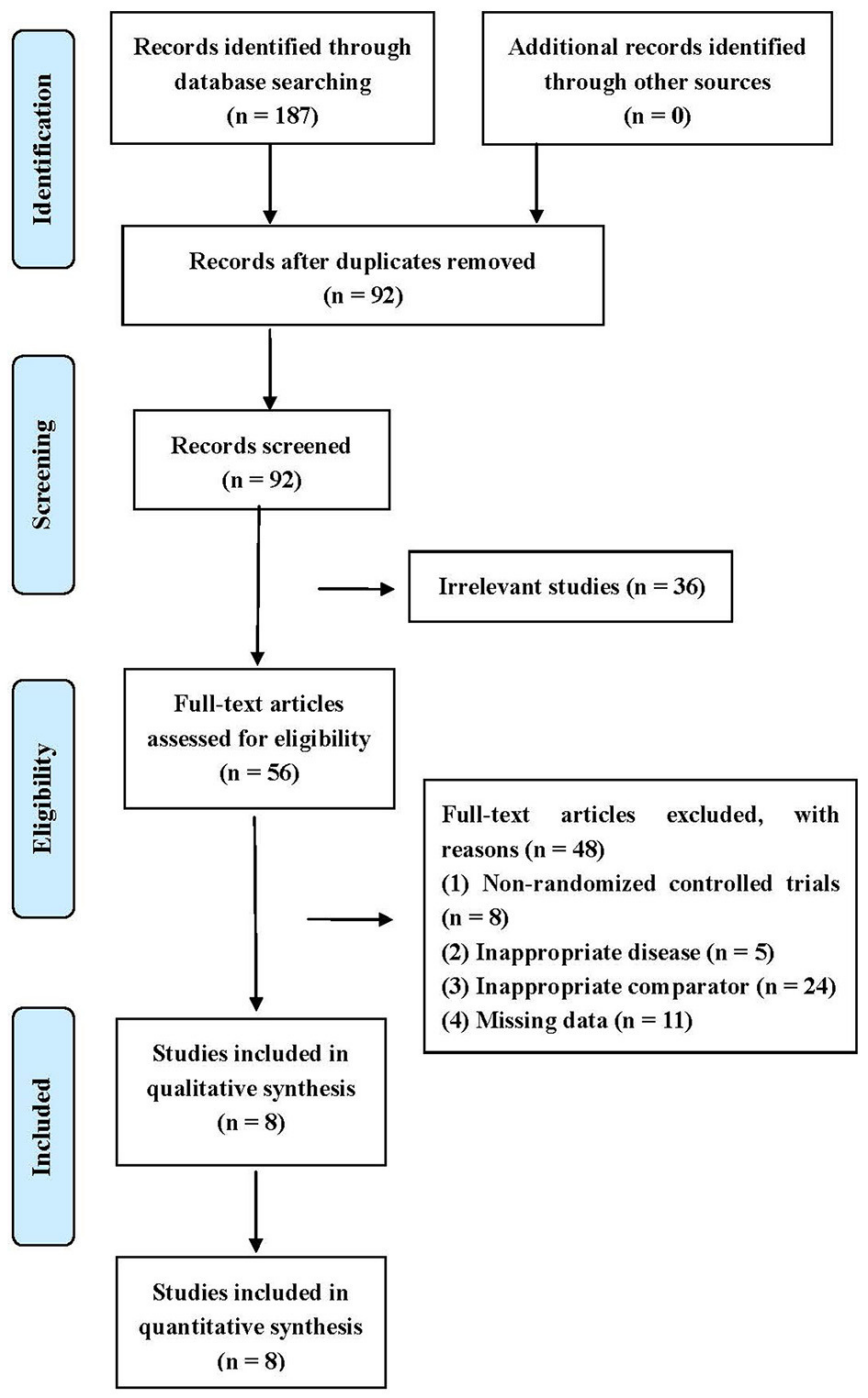
Flow diagram of literature search.

A total of 187 articles were identified in the initial search. After excluding duplicates, 92 articles remained. Thirty-six irrelevant articles were subsequently eliminated after screening titles and abstracts. Full texts of the remaining articles were assessed for eligibility. Finally, eight studies were included for statistical analysis (19, 23–29). The screening process is shown in Figure 1.

### Study Characteristics

The characteristics of included studies are presented in Table 1. To be specific, the eight studies were published from 2008 to 2019, involving 618 participants (322 in the experimental group and 296 in the control group). The sample size ranged from 20 to 45 in each group. Six studies used Chinese Classification of Mental Disorders (CCMD) to diagnose insomnia. One study used ICD-10 and CCMD-3 to diagnose insomnia. Another one used DSM-IV-TR to diagnose insomnia. The interventions in the experimental group included AA, AA plus routine nursing care, and AA plus footbath. The interventions in the control group included sham AA, estazolam, routine nursing care, and footbath. Duration of treatment ranged from 4 weeks to 2 months. The indicators of outcome included PSQI score, response rate, use of sleep medications, and adverse reactions.

**Table 1:** Characteristics of included studies.

**Article**	**Diagnostic criteria for insomnia**	**Sample size in the experimental group**	**Sample size in the control group**	**Intervention(s) in the experimental group**	**Intervention(s) in the control group**	**Frequency of AA**	**Duration of treatment**	**Outcomes**
Zhang (23)	ICD-10,CCMD-3	20	20 in each control group	AA	Estazolam in control group (1); mental health education in control group (2)	Four to five times a day	8 weeks	PSQI, Response rate
Zhao (24)	CCMD-3	30	30	AA	Estazolam	Four times a day	8 weeks	Response rate
Zhou et al. (25)	CCMD-3	30	30	AA	Estazolam	Four to five times a day	1 month	PSQI, Response rate
Zheng and Liu (26)	CCMD-2	40	40	AA	Estazolam	Not reported	8 weeks	PSQI
Zou et al. (19)	DSM-IV-TR	32	31	AA	Sham AA	Three to five times a day	8 weeks	PSQI, Response rate, Use of sleep medications, adverse reactions
Yin (27)	CCMD-2	45 in each experimental group	45	AA in experimental group (1); AA + foot bath in experimental group (2)	Foot bath	Not reported	4 weeks	PSQI, adverse reactions
Huang et al. (28)	CCMD	40	40	AA	Estazolam	Four to five times a day	2 months	Response rate
Li and Zhang (29)	CCMD	40	40	AA + routine nursing care	Routine nursing care (health education, emotional support, etc.)	Three to five times a day	4 weeks	PSQI, Response rate

*ICD-10, International Classification of Diseases (10th edition); CCMD-2, Chinese Classification of Mental Disorders (2nd edition); CCMD-3, Chinese Classification of Mental Disorders (3rd edition); DSM-IV-TR, Diagnostic and Statistical Manual of Mental Disorders fourth edition-Text Revision; AA, auricular acupressure; PSQI, Pittsburgh Sleep Quality Index*.

### Quality Assessment

**Figure 2:**
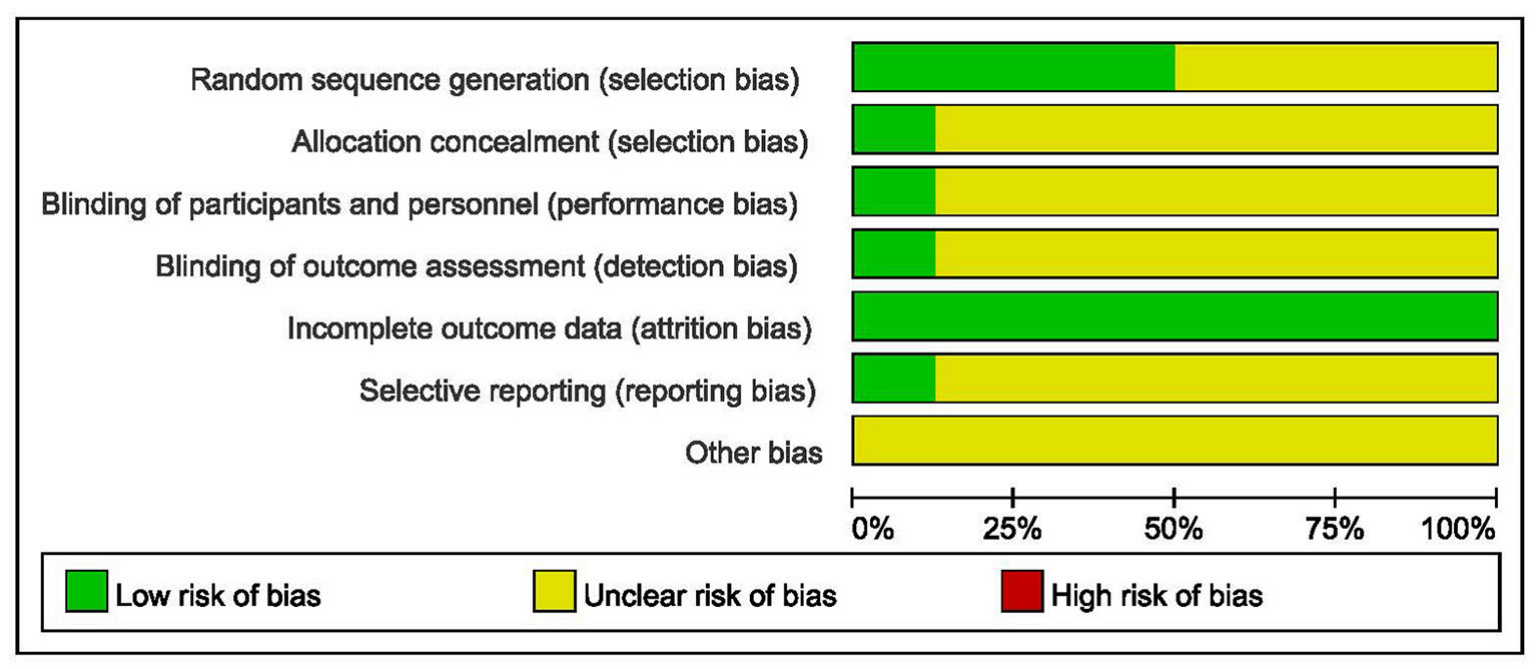
Risk of bias graph.

**Figure 3:**
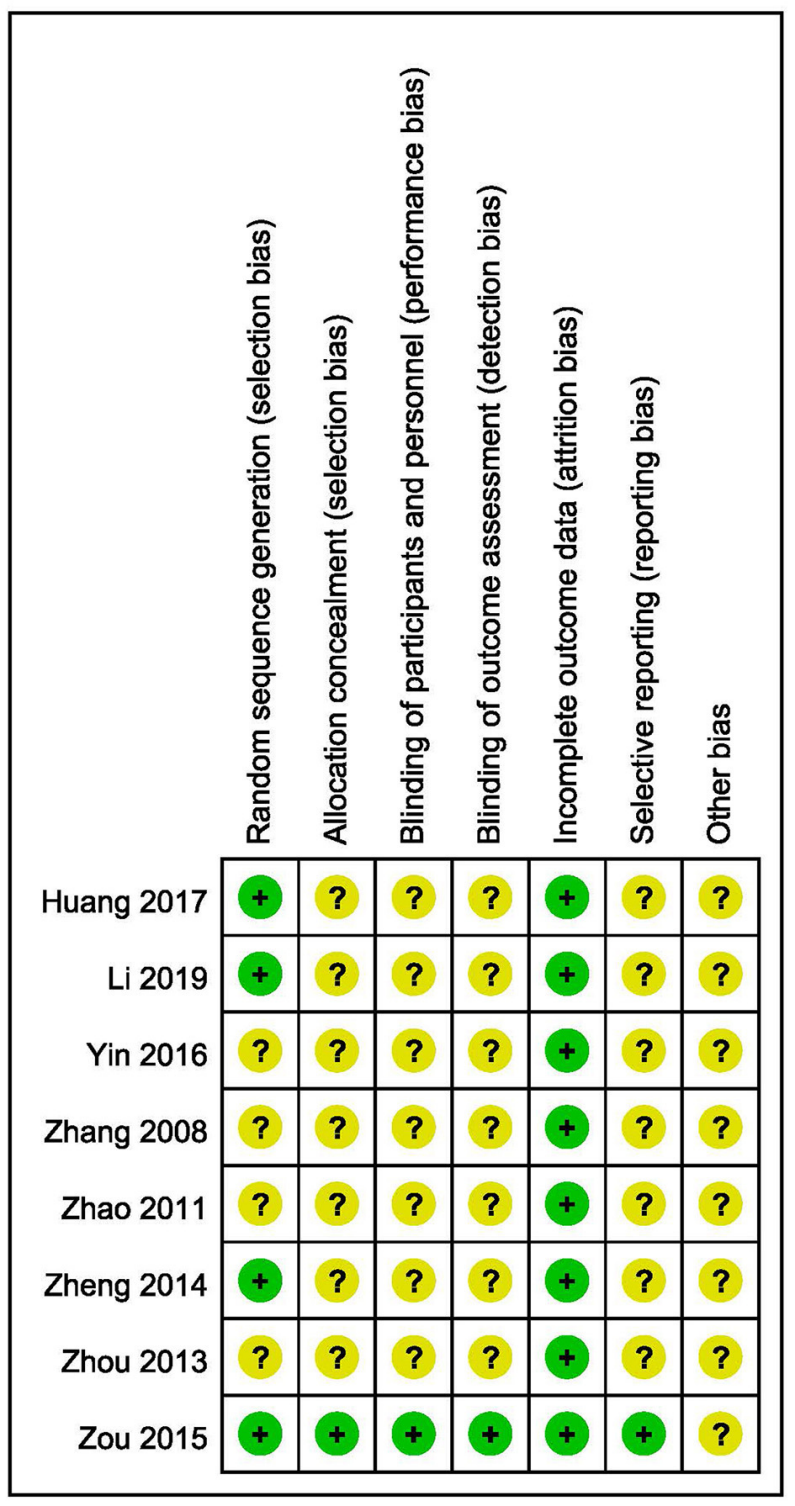
Risk of bias summary.

The outcomes of risk of bias assessment are summarized in Figures 2, 3. A random number table was used for participant assignment in four of the included studies (19, 26, 28, 29), while the specific randomized allocation method was not described in other studies. Only one study reported allocation concealment and blinding (19). Complete outcome data were available in all studies. One study was graded low risk of bias in reporting bias (19). The risk of bias in other bias was graded unclear in all included studies.

### PSQI Score

Six included studies reported PSQI global score (19, 23, 25–27, 29). Due to the heterogeneity of therapeutic duration and comparisons, the subgroup or narrative analysis was conducted instead of the meta-analysis including all eligible studies.

**Figure 4:**
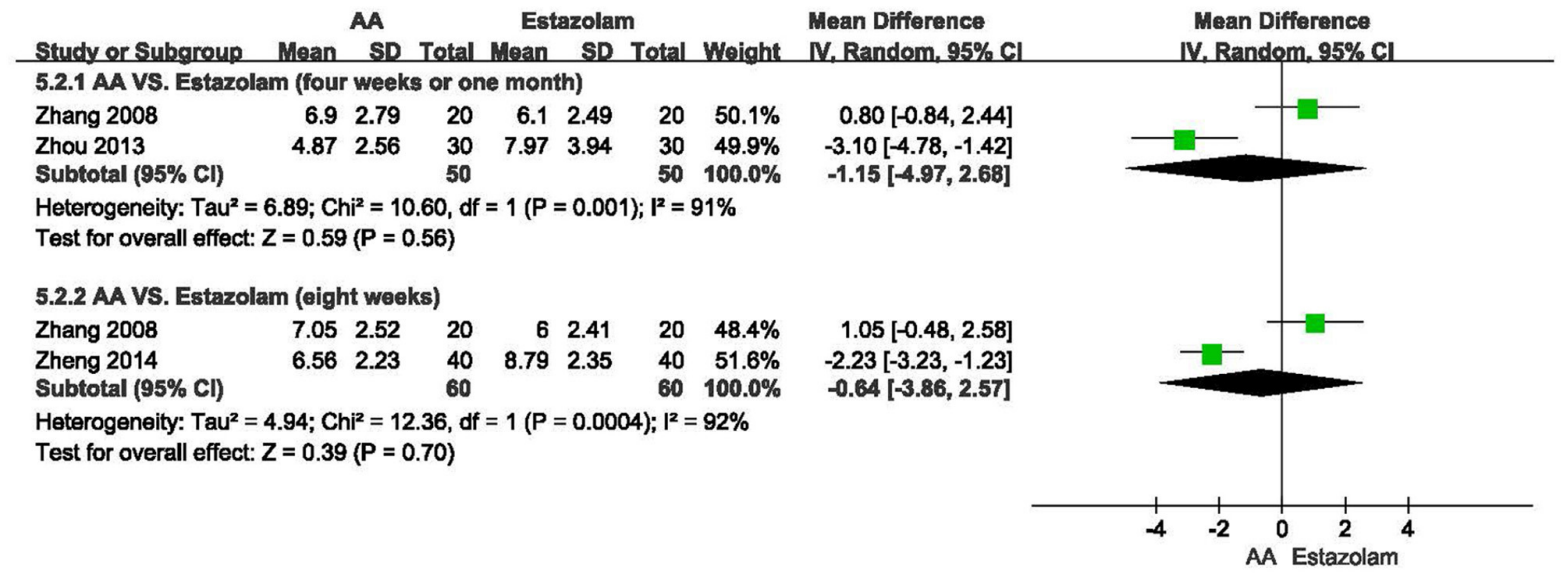
Meta-analysis of AA vs. estazolam on PSQI global score.

As shown in Figure 4, there was no significant difference of PSQI global score after 4 weeks or 1 month of AA treatment compared with estazolam (*n* = 100, MD = −1.15, 95% CI: −4.97 to 2.68, *p* = 0.56), which was similar with the outcome of meta-analysis at the end of 8 weeks (*n* = 120, MD = −0.64, 95% CI: −3.86 to 2.57, *p* = 0.70).

One study found that AA alone could significantly attenuate the PSQI global score compared with mental health education alone (*n* = 40, MD = −1.80, 95% CI: −3.41 to −0.19, *p* = 0.03, duration of 4 weeks; *n* = 40, MD = −1.80, 95% CI: −3.26 to −0.34, *p* = 0.02, duration of 8 weeks) (23). Another study revealed that the PSQI global score was significantly decreased after 4-week administration of AA plus routine nursing care compared with routine nursing care alone (*n* = 80, MD = −2.38, 95% CI: −3.46 to −1.30, *p* < 0.0001) (29). Moreover, a three-arm clinical trial was conducted to assess the efficacy of AA, footbath, and a combination of both for insomnia in MHD patients (27). As a result, there was no significant difference between AA and footbath (*n* = 90, MD = 0.36, 95% CI: −0.92 to 1.64, *p* = 0.58). However, AA combined with footbath could significantly attenuate the PSQI global score in comparison with footbath alone (*n* = 90, MD = −1.36, 95% CI: −2.41 to −0.31, *p* = 0.01). A pilot trial found that AA could decrease PSQI global score compared with sham AA, but without statistical significance (*n* = 63, MD = – 1.55, 95% CI: −3.56 to 0.46, *p* = 0.13) (19).

### Response Rate

Among the eight included studies, six of them reported the response rate (19, 23–25, 28, 29). The definition of response was various across these studies.

**Figure 5:**
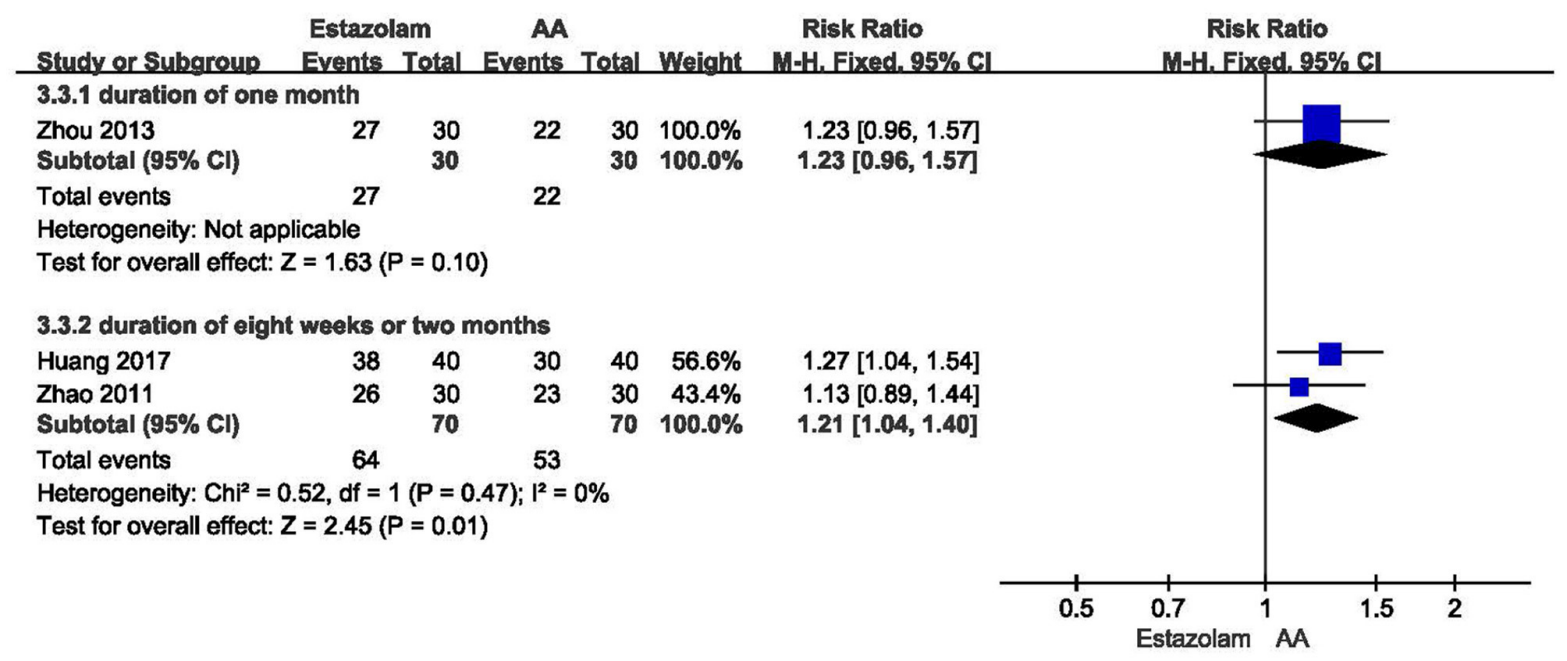
Meta-analysis of AA vs. estazolam on response rate.

In three trials, response was defined as reduction of PSQI global score by 25% or more (24, 25, 28). One study found no significant difference of response rate after 1-month treatment between AA and estazolam (*n* = 60, RR = 1.23, 95% CI: 0.96 to 1.57, *p* = 0.10, Figure 5) (25). The meta-analysis including two studies showed that AA alone could significantly increase response rate compared with estazolam alone at the end of 8 weeks or 2 months (*n* = 140, RR = 1.21, 95% CI: 1.04 to 1.40, *p* = 0.01, Figure 5).

In another study, response was defined as reduction of PSQI global score by 20% or more (29). As a result, AA plus routine nursing care could significantly improve response rate in MHD patients compared with routine nursing care alone (*n* = 80, RR = 2.20, 95% CI: 1.44 to 3.36, *p* = 0.0003). Another study defined response as reduction of PSQI global score by five points and more (23), which showed that AA could significantly increase response rate compared with mental health education (*n* = 40, RR = 1.50, 95% CI: 1.02 to 2.21, *p* = 0.04), However, no statistical difference of response rate was identified between AA and estazolam (*n* = 40, RR = 1.00, 95% CI: 0.81 to 1.23, *p* = 1.00). Another trial defined response as reduction of PSQI global score by three points and more (19), which revealed that the response rate was significantly higher in the AA group than the sham AA group (*n* = 63, RR = 1.94, 95% CI: 1.09 to 3.45, *p* = 0.02).

### Use of Sleep Medications

One study reported that the proportion of patients taking sleep medications was significantly reduced (*p* < 0.01) in the AA group, compared with the sham AA group after 8 weeks of treatment (19).

### Adverse Reactions

Two studies described the adverse reaction. One study showed no adverse reactions in the AA or sham AA group (19), while another study revealed no adverse reactions in the AA plus footbath or footbath alone group (27).

### Publication Bias

Publication bias was not assessed because no meta-analyses included over 10 studies.

## Discussion

### Main Findings and Interpretation

In this study, we critically evaluated the efficacy and safety of AA for insomnia in MHD patients.

A pilot study showed no statistical difference in PSQI global score, but significant difference in response rate between AA and sham AA (19). Power is defined as the probability of rejecting a false null hypothesis (30). It is generally assumed to be 80% to 90% when calculating sample size (30). In this pilot study, however, the sample size was not calculated. Herein, we calculated the power of these two indicators using the results by PASS software, which showed a power of 32 and 67%, respectively, to detect a superiority margin difference between AA and sham AA. They might be insufficient to reject a false null hypothesis. Therefore, high-quality trials with a larger sample size are further warranted.

A clinical practice guideline in 2017 reported that estazolam (a benzodiazepine derivative) could significantly improve sleep duration compared with placebo (9). Estazolam has also been approved to treat insomnia in the United States by the Food and Drug Administration (7). However, a study found that benzodiazepines were associated with risk of any fracture (RR = 1.31, *p* = 0.03) in hemodialysis patients (31). Another study demonstrated that benzodiazepines may be associated with greater mortality in incident dialysis patients (32). Our meta-analyses showed no significant difference of PSQI global score between AA and estazolam. However, these meta-analyses had some defects, such as high statistical heterogeneity (*I*^2^ > 90%), small sample size, and a small number of included studies. We found that one study with a small sample size reported no significant difference of response rate after 1-month treatment between AA and estazolam. However, a meta-analysis involving another two studies showed that response rate was significantly higher after 8 weeks or 2 months of AA treatment compared with estazolam. One study reported that AA of 8 weeks' duration could significantly improve patient comfort level measured by Maintenance Hemodialysis Patients Comfort Scale compared with estazolam (24). No significant difference or inconsistent results may be associated with multiple factors, such as severity of insomnia, small sample size, and cumulative effect. It is important to use double-blind and double-dummy technology in trials comparing two interventions, which can reduce performance bias, detection bias, etc. However, only one study reported blinding (19). Double-dummy technology was not reported in included studies. AA includes multiple steps, such as selecting acupoints, taping drugs or magnetic pellets, and physical pressure. These steps may be various across included studies. For example, Huang et al. taped semen vaccariae on patients' ears (28). Magnetic pellet was used in another study (24). They may partly explain the above results. The safety of AA vs. estazolam for insomnia in MHD patients was not assessed due to a lack of information. In addition, the efficacy and safety of AA vs. other drugs for insomnia in MHD patients were also not investigated due to the absent trials on this topic.

Some clinical guidelines reported that psychological and behavioral therapies were used for the management of insomnia (7, 33). In this review, only one enrolled study found that AA combined with routine nursing care (a combination of health education, emotional support, etc.) could significantly relieve insomnia symptoms in MHD patients in comparison with routine nursing care alone (29). Several previous studies reported that footbath could improve sleep quality (34, 35). One included study showed that AA combined with footbath could significantly attenuate the PSQI global score compared with footbath alone (27), indicating that a combination of AA and routine nursing care/footbath may be a beneficial therapeutic strategy for insomnia management in MHD patients. However, the efficacy of AA combined with drugs such as estazolam for insomnia in MHD patients was not assessed due to a lack of relevant trials in this study.

### Limitations

Several potential limitations should be taken into consideration. To begin with, a small sample size may decrease the probability of rejecting a false null hypothesis. Secondly, performing the meta-analysis was limited due to the heterogeneity of duration, definition of response rate, etc. Thirdly, the reported outcome measurement varied across studies. No core outcome set on this topic was published in Core Outcome Measures in Effectiveness Trials (COMET). Therefore, it is necessary to establish a core outcome set on this topic. Fourthly, the methodological quality of included studies may be reduced due to unclear risk of bias of allocation concealment and blinding item in most of the included studies. Fifthly, only two studies reported adverse reactions. Therefore, the evidence is insufficient to evaluate the safety of AA for insomnia in MHD patients. Finally, the causes of insomnia were unrestricted in this study. Only one study reported the causes of insomnia, but did not provide the efficacy of AA according to different causes of insomnia (23). Therefore, no subgroup analysis based on causes of insomnia was conducted because of insufficient information.

## Conclusion

The present findings in our study suggest that AA may be an alternative treatment for insomnia in patients with MHD. However, more large-scale, high-quality trials are still warranted to confirm these outcomes.

## Data Availability Statement

The original contributions generated for this study are included in the article/supplementary material, further inquiries can be directed to the corresponding author/s.

## Author Contributions

JZ, MP, and HY conceived the study. JC designed the search strategy. JC and MP screened the studies. BY and KY extracted the data. SD and MP assessed the methodological quality. LW performed the statistical analysis. MP, JC, SD, BY, KY, LW, JZ, and HY drafted the manuscript and reviewed and revised the manuscript. All authors have read and approved the final version of the manuscript.

## Conflict of Interest

The authors declare that the research was conducted in the absence of any commercial or financial relationships that could be construed as a potential conflict of interest.
